# Pushing Boundaries in Aesthetic Breast Surgery: Precise Surgical Planning With Online Consultation and 3-dimensional Simulation

**DOI:** 10.1097/GOX.0000000000007203

**Published:** 2025-11-10

**Authors:** Carlotta Barbon, Per Hedén, Maximilian Otte

**Affiliations:** From the * University of Zurich, Zurich, Switzerland; † University Hospital of Zurich, Zurich, Switzerland; ‡ The Faculty, Stockholm, Sweden.

## Abstract

**Background:**

In the past decade, there has been an increase in the implementation of telemedicine and 3-dimensional (3D) imaging in plastic and reconstructive surgery. This retrospective study assessed the feasibility of precise surgical planning for breast procedures and the conversion rate of online consultations with 3D imaging for surgeons who work in multiple countries.

**Methods:**

Fifteen consecutive patients underwent online visits with 3D imaging for breast augmentation (10), hybrid augmentation (2), mastopexy (2), and breast reduction (1). All patients were physically seen for the first time the day before the operation; however, the surgical plan and the types of implants were decided with the online consultation. The conversion rate was compared with that of 30 consecutive patients seen in the same timeframe in the regular outpatient clinic.

**Results:**

The procedures could be performed as foreseen. The implants chosen were accurate in 100% of the patients; only 1 implant per breast could be ordered for 80% of patients and 2 for the other 20%. The patients were effortlessly followed up with online consultations and expressed satisfaction with the results. The conversion rate was 87% for the online consultations compared with 90% for the regular encounters (*P* = 0.74).

**Conclusions:**

Online consultations with 3D imaging transcend geographic limitations by providing effective result visualization and allowing for accurate planning, without affecting the conversion rate. Considering limited real-world data, our findings help fill a key gap and support broader adoption in global plastic surgery.

**Takeaways**

**Question:** Can online consultations with 3-dimensional (3D) imaging achieve accurate surgical planning and maintain conversion rates in international plastic surgery practice?

**Findings:** In this retrospective study, 15 patients underwent breast procedures planned entirely through online 3D consultations. All surgical outcomes matched the preoperative plans with 100% implant accuracy and no intraoperative changes. The conversion rate (87%) was comparable to in-person visits (90%, *P* = 0.74).

**Meaning:** Online 3D consultations enable precise, cross-border surgical planning in aesthetic breast surgery without compromising accuracy or conversion rates.

## INTRODUCTION

In recent years, plastic and reconstructive surgery has seen increased use of telemedicine. This trend has been slowly developing over the years, with a significant boost during the COVID-19 pandemics.^1^ Moreover, the rise of accessible video tools (eg, Zoom, Teams, WhatsApp, FaceTime) has considerably increased demand for online consultations.

In a field like breast surgery, online consultations are dependent on reliable methods that perform precise measurements; 3-dimensional (3D) imaging provides a convenient, objective, and reproducible tool that is indispensable for the surgeon.^2–4^ It also helps patients visualize results, aligning expectations for volume and shape while predicting outcomes.^5,6^

Social media have dismantled geographic barriers, giving patients unprecedented access to surgeons across borders—unthinkable just a decade ago. The author of this study (M.O.), who practices in multiple clinics across 2 countries, naturally adopted this approach to overcome the limitations imposed by physical distance. Overall, there is limited research on this topic, especially on the conversion rate of virtual visits, which is the number of patients undergoing surgery with respect to the ones who performed a consultation. One could believe that in-person visits have more successful conversion rates; however, a study demonstrated that patients were just as likely to undergo surgery, regardless of the type of encounter.^7^

This virtual implementation is surely not an answer for everyone, both patients and surgeons must feel confident; certain populations have more difficulties in transitioning to telemedicine, in part because of lack of acquaintance with technology and partly because of a personal predilection for an in-person interaction; nevertheless, online consultations significantly broaden the possibilities for both the patients and the surgeons.

## METHODS

We present 15 consecutive patients who underwent online consultations for aesthetic breast surgery. To help patients visualize the results and for the surgeon to select the proper implants, virtual 3D computer simulations have been performed in all the patients. The software used is Arbrea Breast Software (ABS), developed by a Swiss-based company in Zurich that specializes in 3D live augmented reality. This tool runs directly on an iPhone or iPad, using the device’s built-in artificial intelligence. Importantly, data are processed directly on the mobile device (edge computing) and do not need to be transferred to a cloud to be processed. Thus, the pictures do not have to leave the device, unless it is decided from the patient and the surgeon. This not only ensures full security and privacy of patients’ data, but also makes the processing efficient, flexible, and fast; within seconds the image is reproduced on the device.

**Fig. 1.**
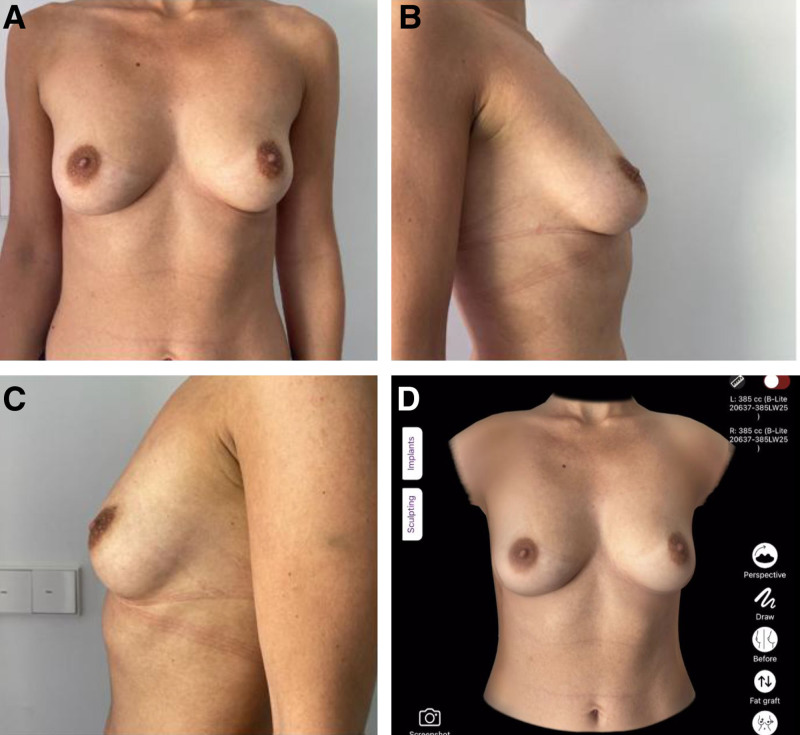
Images needed for the 3D simulation. Three images, 1 front (A) and 2 lateral (B, C) views, are provided by the patients, or alternatively, screen shots can be taken from the surgeon during the online consultation. The fourth image (D) shows how the software reproduces the patient’s breast and chest wall.

**Fig. 2.**
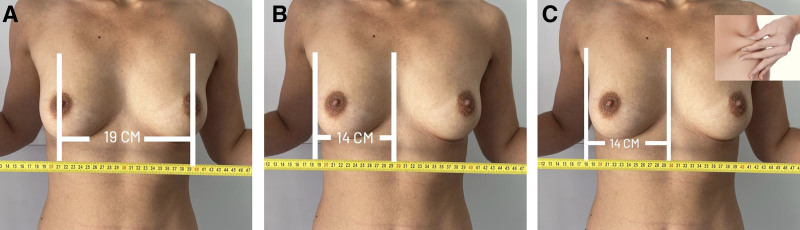
Images needed for the 3D simulation. A–C, The patient measures the nipple distance and breast width with a tape measure. The pinch test must be estimated by the surgeon, based on clinical experience.

**Fig. 3.**
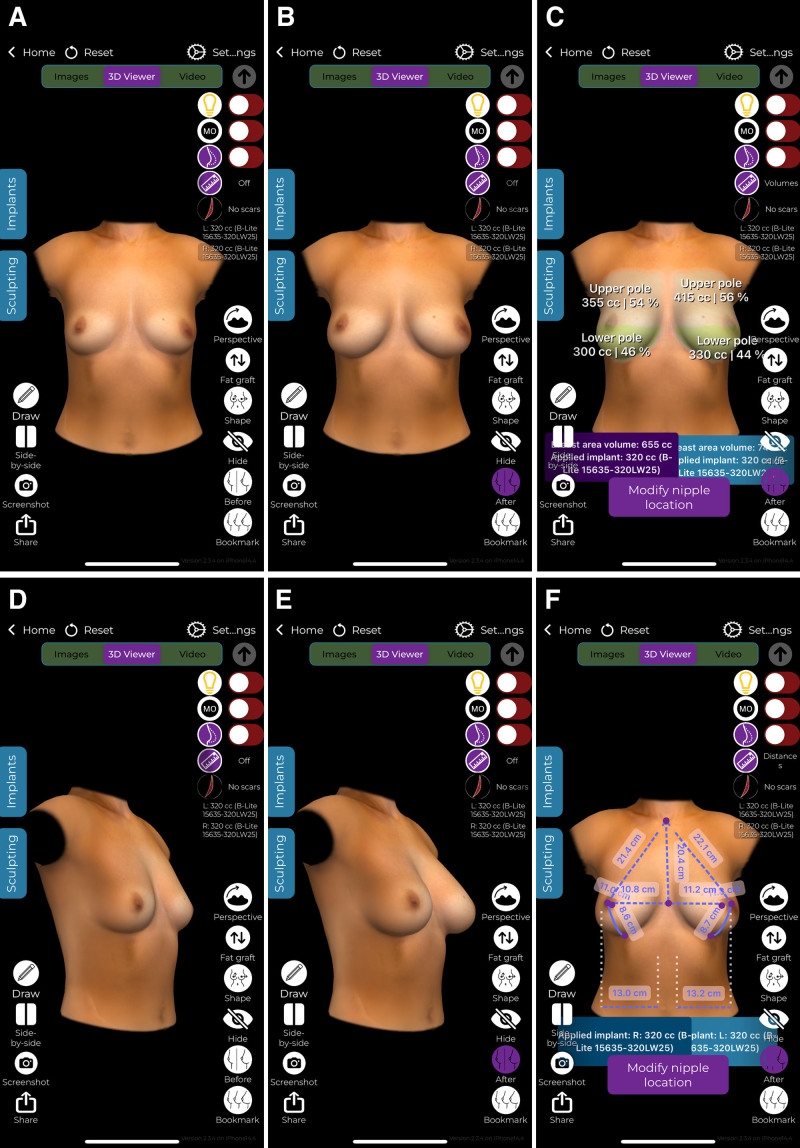
Images needed for the 3D simulation. A, Frontal view of a preoperative 3D reconstruction on the application without implants. B, Frontal view of a simulation with a 320-mL B-Lite implant. C, Calculation of the total breast area volume with the applied implant of 320 mL. D, Side view of a preoperative 3D reconstruction on the application without implants. E, Side view of a simulation with a 320-mL B-Lite implant. F, Breast measurements provided by the application.

The surgeon began by capturing 3 patient images using the iPad’s standard camera; alternatively, these can be sent by the patient before the online consultation (1 front and 2 lateral views) (Fig. 1). To generate accurate 3D simulations and select the appropriate implant, patients were instructed to perform 2 key measurements at home using a flexible measuring tape. Nipple-to-nipple distance was measured horizontally from the center of 1 nipple to the other. The other parameter is breast width, measured horizontally across the base of each breast, from the medial to lateral border. These values were entered into the ABS application by the surgeon to enhance the accuracy of the 3D model. Importantly, the surgeon assisted the patient during the video consultation in performing the measurements. As shown in Figure 2, the patient could hold the tape below the breasts while the measurements were taken with the surgeon’s guidance. The pinch test was visually estimated by the surgeon during the video consultation, based on clinical experience and observation of upper pole contour and body habitus; it was not performed by the patient, as we considered it unreliable due to variability in execution, positioning, and pressure. This estimation served to guide implant selection and surgical planning. Thereafter, the software on the iPad, without reliance on external servers or internet connectivity, rapidly generated a comprehensive 3D simulation of postoperative results (Fig. 3). This approach prioritized privacy and expedited the process. This software displayed 3D simulations and breast measurements from multiple perspectives. It offered a choice between a constructed 3D view from 3 images or real-time augmented reality projections directly onto the patient’s body. These diverse viewing capabilities allowed patients to assess different implant types, sizes, and shapes, providing a clearer understanding of potential outcomes. A noteworthy advantage was the software’s speed, as it completed the average simulation in 90 seconds.^5^ The patients undergoing breast augmentation had also been advised to simulate the results and feel the weight of the implants, by either placing a stocking filled with rice in their bra, where 140 g corresponded to approximately 1 cup size, or buying online silicone insets.

Using the tape measurements and pinch test estimate, the surgeon could precisely choose the implant. The pinch test, which simulates the patients’ own tissue cover, was then subtracted to better estimate the desired implant size.

To add comfort and freedom, all the patients who were seen for the online consultation were given the option of opting out of surgery with full reimbursement at any time. Likewise, it was communicated to the patient that the surgeon could also decide to opt out of surgery if he felt the procedure could not be completed as anticipated. The patients were seen once they arrived in Stockholm the day before surgery to address any concerns and issues.

Electronic records were analyzed after obtaining written patient consent. Data were then analyzed using Microsoft Excel version 16.6 (Microsoft Corp., Redmond, WA).

To compare the conversion rates, the 15 online consultations were compared with those of 30 consecutive patients from the outpatient clinic seeking the same type of operations during the same period. Of note, the patients were operated on in accordance with the Swedish law, where a minimum of 1 week should pass between the first consultation and the surgery. Patients in the control group also underwent a 3D simulation during their in-person consultation using the same ABS, ensuring consistency in implant selection and result visualization between both groups.

## RESULTS

Fifteen consecutive patients have been requesting an online consultation for aesthetic breast surgery. All the patients were women; 12 requested an augmentation, 1 a mastopexy, and 2 a reduction in mammoplasty. Of these, 40% of the patients did not live in Sweden. Among the patients who requested an implant, 73% decided to use either the rice or the silicon insets to simulate the weight and the contour of the implants (Table 1).

**Table 1.** Patients’ and Surgery Characteristics

	Online Consultations		In-patient Consultations
Patients (N)	15		30
Age (mean ± SD), y	32 (±6)		36 (±9)
BMI (mean ± SD), kg/m^2^	23 (±3)		23 (±3)
Patient’s residency, n (%)			
In the same country	9 (60%)		27 (90%)
In another country	6 (40%)		3 (10%)
Length of consultation (mean ± SD), min	31 (±6)		32 (±4)
Patients used rice, n (%)	11 (73%)		N/A
Patient also seen in clinic, n (%)	4 (27%)		N/A
Conversion rate, n (%)	13 (87%)	27 (90%)	
Surgery performed, n (%)			
Augmentation	10 (77%)		13 (48%)
Reduction	1 (8%)		10 (37%)
Mastopexy	2 (15%)		4 (15%)
Ordered implants correct, n (%)	10 (100%)		N/A
Average implant (mean ± SD), cc	281 (±47)		294 (±66)

N/A, not applicable.

Among the 15 patients who underwent the first consultation online, 13 decided to undergo surgery (87%). Whereas among the 30 patients who have been seen in the clinic, the conversion rate was 90%, (*P* = 0.74) (Table 1).

The operations performed on the patients who underwent online consultations were augmentation in 77% of patients (2 of these patients underwent hybrid augmentation with implant and fat augmentation), mastopexy in 15%, and reduction in mammoplasty in the remaining 8% (Table 1). No patient decided to cancel the surgery.

Regarding the breast augmentations, one implant per breast was ordered for 80% of the patients, and two implants per breast were ordered for the remaining 20%. The implants used were anatomic (70%) and round (30%); the mean volume was 281 mL (±47 mL). All the operations could be performed smoothly, and the implant chosen with the virtual consultations was correct in 100% of the cases, and all the patients demonstrated full satisfaction with the selected implants and the results.

## DISCUSSION

We present a series of patients who underwent aesthetic breast surgery in which the surgical planning and the implant choice were made solely through an online consultation using 3D imaging. Although research on online consultations remains limited, interest in the topic has grown significantly since the COVID-19 pandemic. To the best of our knowledge, this is the first article on online consultations with 3D imaging as the sole organizational encounter before aesthetic breast operations. Although previous studies have broadly evaluated telemedicine in surgical care, little is known about its application in fully remote preoperative planning for aesthetic procedures. Data on conversion rates and outcomes following virtual-only consultations remain scarce, and our study aimed to address this gap.

One study performed in 2020 on 374 patients undergoing surgery for benign and malignant breast disease reported that 96 (25.7%) patients had telemedicine consultations, and among these patients, only 39.6% needed an additional in-person visit before their operative date. Of these patients, 45 underwent breast-conserving therapy; 41, mastectomy (25 of which with reconstruction); 2, axillary dissections; and 8, excisional biopsies. All procedures were completed with no changes in surgical plans. This study has a different patient population from ours, given that the patients had breast disease and were not seeking aesthetic breast surgery with implants.^8^ Geographic barriers have diminished, thanks to digital tools, allowing patients to reach surgeons across borders. For the operating surgeon in this study, who practices internationally, this model became a practical necessity. One of the most critical considerations when approaching this vanguard technique is how precise and accurate 3D imaging is. Variations in the volume, shape, and contour play a crucial role in the strategic considerations for breast procedures, affecting the ultimate visual outcome and patients’ satisfaction.^9^ It is paramount to have a tool that can effectively deliver realistic outcomes and provide precise measurements so that the operator can plan the surgery and order the implants without having to see the patient. The last generation of 3D imaging has been dedicated to standardizing topographic measurements to advance surgical planning and enhance outcomes.^10^ Our experience was extremely positive, and the results mirror the accuracy of these tools. All the procedures have been performed as planned, and the surgeon was able to order only 1 breast implant per breast for 80% of the patients; he would have not chosen different implants in a regular clinic visit. The authors would also like to highlight that once the patients’ measurements are correct, the implants can be safely selected before surgery, without the need to order multiple implants or sizers. Patients were offered in-person visits if desired, but none of these altered the surgical plan established during the online consultation.

The ABS for 3D simulation for preoperative planning of breast augmentation has been previously validated by La Padula et al,^5^ who found strong resemblances between the computerized simulations and the actual postsurgery results. The authors assessed the accuracy of the simulations by conducting a prospective study on patients undergoing breast augmentation, in which the patients were asked to assess the similarity of the visualized breast on the Breast Simulation Assessment scale. The scores ranged from 0 to 4 where 0 was totally different and 4 was identical; the mean scores in the results were 3.4 ± 0.3. Remarkably, the patients expressed high levels of contentment with both the virtual simulations and the final breast size and form, with a mean score on the visual analogue scale of 8.2 ± 1.2. Until recently, such software was costly and necessitated substantial investments in equipment, training, and office facilities. Nowadays, intuitive software that can be used with an iPad are being developed; results are very promising, but more studies comparing 3D simulations and postoperative outcomes are needed.^5^

The author (P.H.) has experienced a significant surge in the demand for online consultations, especially because in his many years of practice, he has had an increasing demand for his services abroad. He did not use any 3D software system, and as an alternative, he requested the patients to have a tape ruler, a marking pen, and a caliper available during the consultation, with which the pinch test could be measured. By asking the patient or relative to perform the measurements under his instructions, a very high accuracy in the suitable implant choice was achieved; however, this was not the method used in this study.

Similarly, Donfrancesco et al^6^ found that 86% of 150 patients felt that virtual simulations accurately predicted outcomes. As patient demand for preoperative visualization increases, such tools may also contribute to better decision-making and fewer corrective procedures.^6,10,11^ Despite these positive outcomes, it is important to explain to the patient that the simulations do not guarantee an exact match with the postoperative result; this is a delicate topic and patients should be informed to set proper expectations.

Noteworthy is the impact of such a technology on the conversion rate. On 301 consecutive patients equally divided between conventional consultation and consultation with virtual simulation, Donfrancesco et al^6^ reported an increase in the conversion rate from 67% to 86% after including the 3D simulation. This tool was included in all our patient encounters, and our conversion rate was comparable between virtual and in-person consultations, aligning with the literature, where several studies showed conversion rates comparable or even higher to that of traditional encounters.^7,12,13^

A study by Khalaf et al^7^ conducted on 1889 patients, of whom 254 received virtual visits, showed that patients had an equal likelihood of undergoing surgery, irrespective of the way they interacted with the surgeon. The authors found no statistically significant difference between the 2 groups, with a conversion rate of more than 60% for both. Furthermore, the study also highlighted that online visits had comparable diagnostic accuracy to the office counterpart and that did not hinder the development of robust relationship between the patient and the doctor.^7^

The surgeons’ perspective should also not be underestimated, as not everyone may feel comfortable in scheduling everything online. In an anonymous survey electronically distributed to American facial plastic and reconstructive surgeons, almost half declared that an in-person visit should always follow a virtual encounter.^14^ Further examples are brought by Alba et al^15^ who analyzed telemedicine in the plastic surgery field also considering the provider’s perspective and found that after using video visits, patients’ feedback was notably more positive than that of the healthcare providers in terms of telemedicine’s usage, user-friendliness, quality of interaction, and reliability. Patients also expressed a considerably higher level of comfort in scheduling surgery without an in-person consultation compared with healthcare providers and reported greater comfort levels when it came to virtual physical examinations, even for sensitive body areas such as breasts and genitals.^15^

In our sample, of the 15 patients who had their initial online consultations, 13 chose to proceed with surgery, representing an 87% conversion rate. In contrast, among the 30 patients who attended in-person consultation requiring similar procedures within the same timeframe, the conversion rate was 90%. Although our sample was small, it still allowed us to observe comparable conversion rates between online and in-person consultations. However, the limited sample size did not permit more advanced statistical analyses. The purpose of this study was to explore the feasibility of remote surgical planning, and we view these results as preliminary data supporting further investigation with larger cohorts. The conversion rates observed may also reflect the patients’ level of commitment. Those who pursued online consultations had likely already considered the logistical and financial implications of traveling and had firmly selected their surgeon despite the distance—suggesting a high degree of motivation. Local patients face fewer barriers to attend a first visit and may explore multiple consultations before committing to surgery.

In the postoperative period, virtual visits can also be used for accurately and efficiently detecting complications. Different studies reported on the efficacy of postoperative follow-up with telemedicine, which decreases visit times without decreasing patient satisfaction for those who elect to participate in remote video visits. Other clear advantages include reduced traveling, increased convenience, reduced costs, and improved access for patients.^16–21^ In our study, 80% of the patients in the online study group also performed the follow-up online; surprisingly also 40% of the patients originally seen with office visits opted for an online follow-up because of convenience. Our patients reported satisfaction with the telemedicine experience, consistent with previous studies. A survey performed on 72 patients reported on an almost 100% satisfaction after online postoperative follow-up; although some were initially skeptical, they ultimately expressed high contentment with the treatment received.^19^ Other studies found that identification of postsurgical complications was not hindered by this technology, and 1 series evaluating more than 100 patients followed up with telemedicine after abdominal surgery reported that only 2.8% of the patients required an in-patient consult.^16,20^ Undoubtedly, it should not be underestimated that there could be limitations due to infrastructure capacity, the constraints of physical examinations, and the safeguarding of data during virtual appointments, but with the proper patient selection, telemedicine can be a valuable tool also in the postoperative management.^1^ In particular, the inability to perform a direct physical examination—such as the pinch test—represents a methodological limitation. Nevertheless, in this highly selected group of patients who explicitly requested remote planning, the surgeon’s visual assessment proved to be both feasible and sufficiently accurate for surgical planning. Another limitation is the lack of long-term follow-up, which prevents conclusions regarding the durability of results and patient satisfaction over time. Future prospective studies with larger cohorts and extended observation periods are needed to confirm these findings.

## CONCLUSIONS

Preoperative online consultations with 3D imaging are a viable option for selected patients and surgeons, especially when physical distance is a limiting factor. They expand access to care, reduce logistical barriers and waiting time, and can be integrated into postoperative follow-up when appropriate. Not to underestimate, the conversion rate does not seem to be negatively affected.

Although technical issues and limitations in the physical examination remain, these can often be mitigated by surgeon expertise and careful patient selection. Importantly, patients should always retain the option for in-person evaluation. This study does not advocate replacing traditional visits but illustrates that, in specific cases where online consultations were explicitly requested, accurate surgical planning remained achievable. As plastic surgery becomes increasingly globalized, virtual planning tools—particularly 3D imaging—may complement standard care pathways and offer a mutually beneficial alternative for both patients and providers.

## DISCLOSURE

Dr. Hedén is a speaker for Mentor and Motiva. He is a key opinion leader for both and is compensated for his lectures. Dr. Otte is a speaker for POLYTECH Health & Aesthetics GmbH. He has been compensated for speaker presentations (International Society of Aesthetic Plastic Surgery, Cartagena), launching events (MESMO launch in Shanghai), and online workshop for Aleamed. The other author has no financial interest to declare in relation to the content of this article.
